# Supplementary material for: Ubiquitylation-dependent Rap2 activation regulates lamellipodia dynamics during cell migration
Source: J Cell Sci. 2025 Dec 16;138(23):jcs264375. doi: 10.1242/jcs.264375 (PMC12752515; doi:10.1242/jcs.264375)
Supplement: Supplementary information [file joces-138-264375-s1.pdf]

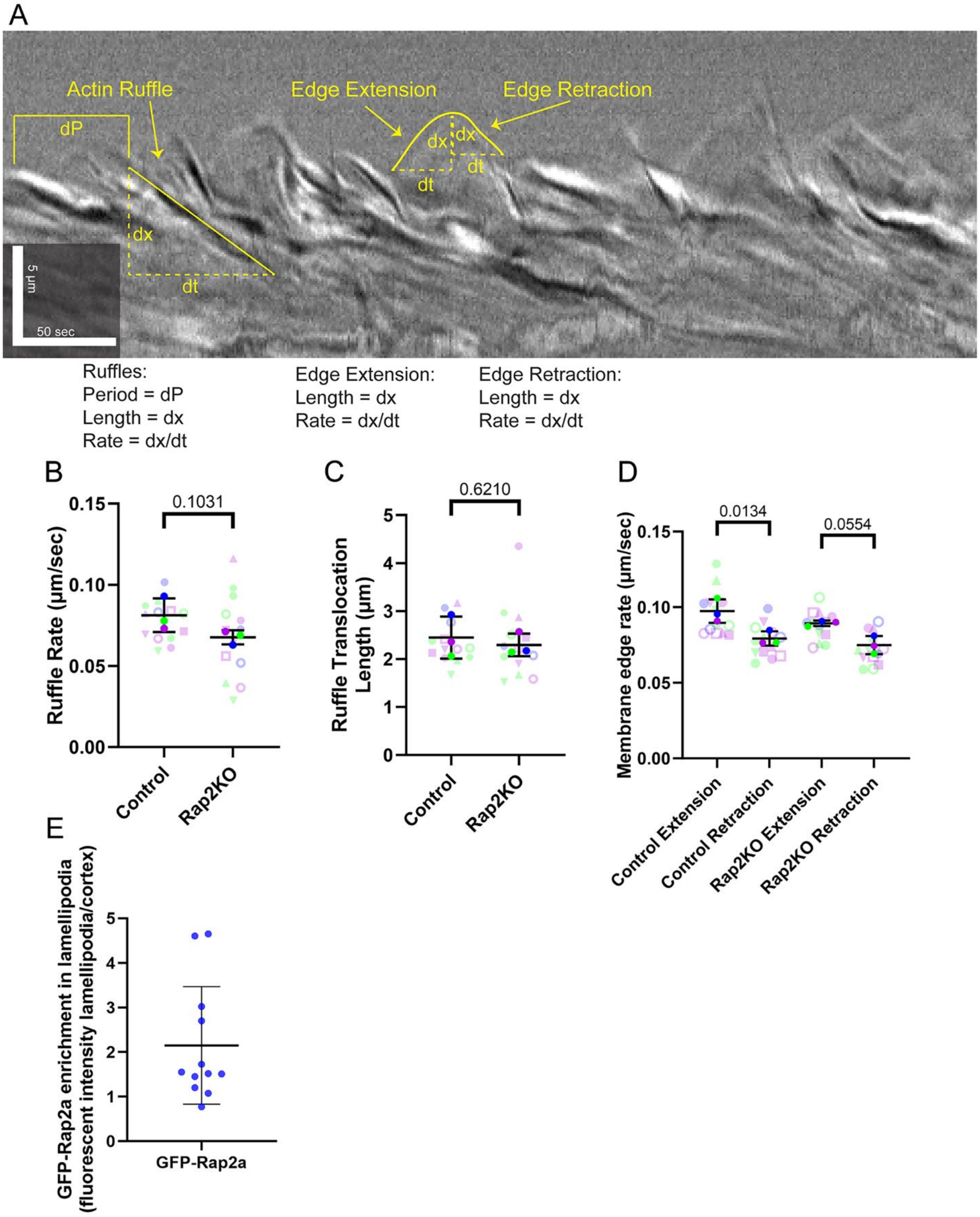


Journal of Cell Science • Supplementary information

**Fig. S1. SACED analysis of the role of Rap2 on lamellipodia dynamics and Rap2 regulation of RhoA activity.**

**(A-D)** Kymographs taken from SACED time-lapse imaging of control and Rap2-KO cells (Videos 3 and 4). Annotated kymograph depicting the different measurements used for SACED calculations **(A).** Quantifications (n=3 biological replicates, control=13 total cells, Rap2-KO=13 total cells. One-tailed student's t-test. Average is mean of biological replicates, error bars represent biological replicate standard deviation) showing actin ruffle rate **(B)** distance of actin ruffle translocation **(C)** and membrane extension/ retraction rates (One-Way ANNOVA with multiple comparisons. Average is mean of biological replicates, errors bars represent biological replicate standard deviation. Significant P-values of vital comparisons shown) **(D).**

**(E)** Quantification of GFP-Rap2a lamellipodium enrichment (n=12 total cells, 27 total lamellipodia)

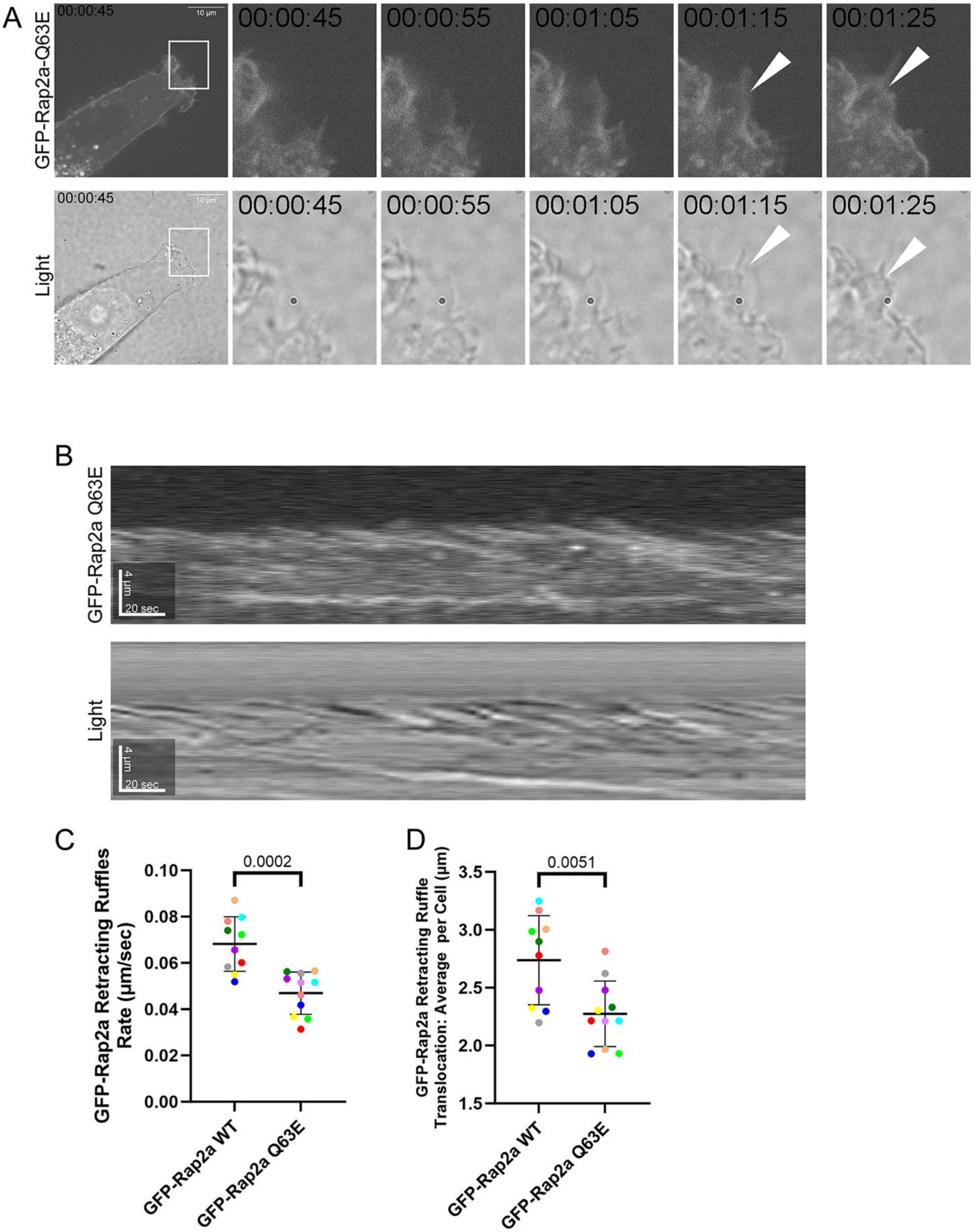


Journal of Cell Science • Supplementary information

**Fig. S2. The Rap2a-Q63E mutation alters lamellipodia ruffling dynamics.**
**(A)** GFP-Rap2-Q63E expressing MDA-MB-231 cells were analyzed by time-lapse microscopy (see Video 11). Shown are selected GFP-Rap2a-Q63E and transmitted light images taken from time-lapse series. White arrow points to retracting ruffle with accumulating GFP-Rap2a-Q63E.
**(B-D)** Kymographs generated from GFP-Rap2a-Q63E time-lapse series (Video 11). Quantification (GFP-Rap2a-WT n=10 cells, GFP-Rap2a-Q63E n=11 cells, ruffles averaged from 2 kymographs per cell. One-tailed student's t-test. Average is mean of biological replicates, error bars represent biological replicate standard deviation) of GFP-Rap2a-Q63E ruffles dynamics as compared to GFP-Rap2a-WT ruffle dynamics (Fig. 1I, Video 5) showing changes in ruffle retraction rate **(C)** and translocation distance **(D)**.

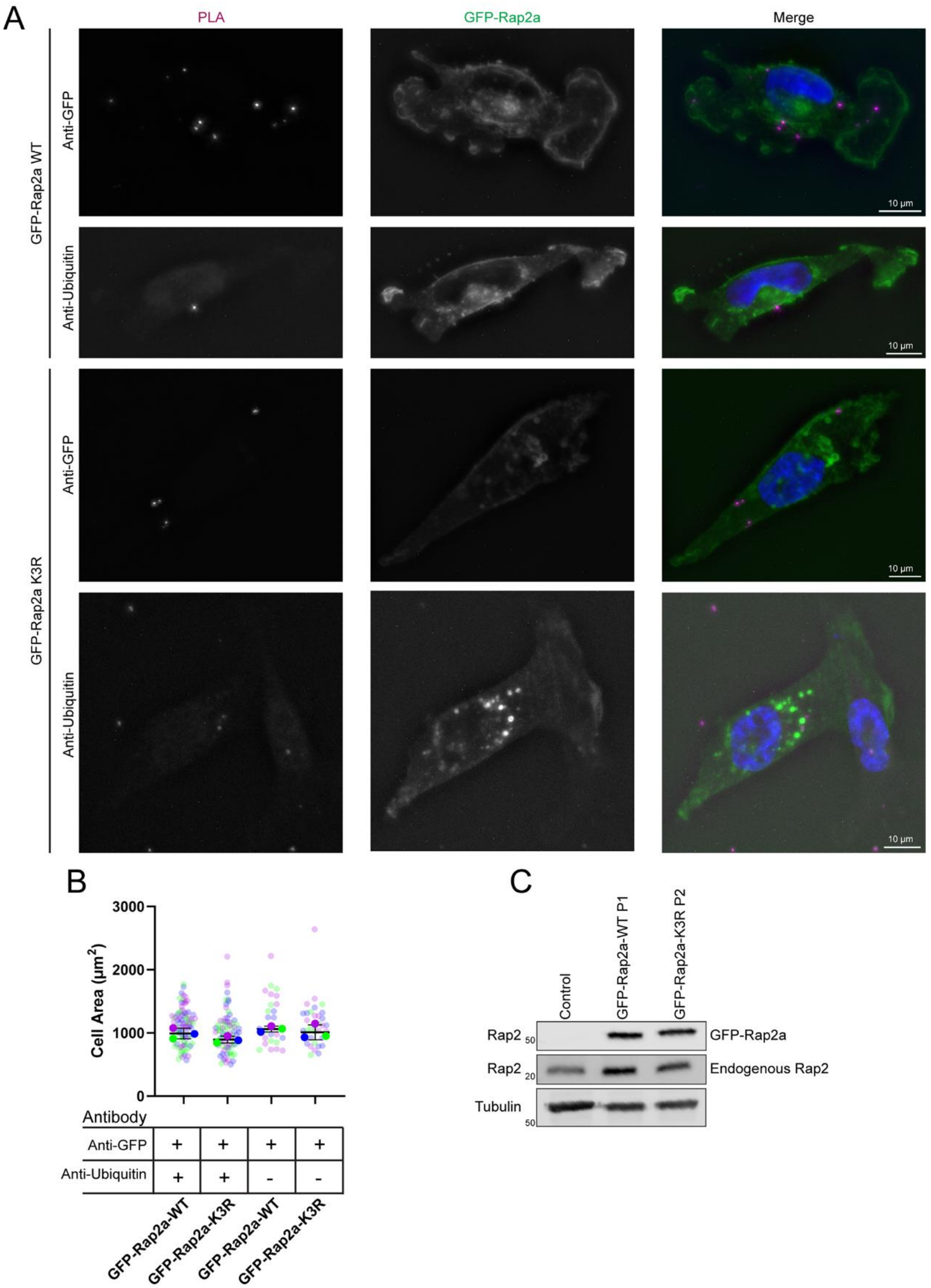


Journal of Cell Science • Supplementary information

**Fig. S3. PLA Controls.**

**(A-B)** Control data from PLA experiments. Panel **(A)** shows PLA experiments performed with only an anti-GFP or an anti-ubiquitin antibody in cell with GFP-Rap2a-WT or GFP-Rap2a-K3R expression. Minimal PLA puncta are observed. Quantification (n=3 biological replicates, GFP-Rap2a-WT PLA=85 total cells, GFP-Rap2a-K3R PLA=76 total cells, GFP-Rap2a-WT GFP control=27 total cells, GFP-Rap2a-K3R GFP control=32 total cells. One-Way ANNOVA with multiple comparisons. Average is mean of biological replicates, errors bars represent biological replicate standard deviation. Significant P-values shown) from PLA experiments showing cell area **(B).**

**(C)** Western blot of GFP-Rap2a-WT and GFP-Rap2a-K3R in early passage cells (WT P1, K3R P2). Increased Rap2 at the endogenous size in GFP-Rap2a expressing cells is as a result of construct cleavage.

Journal of Cell Science • Supplementary information

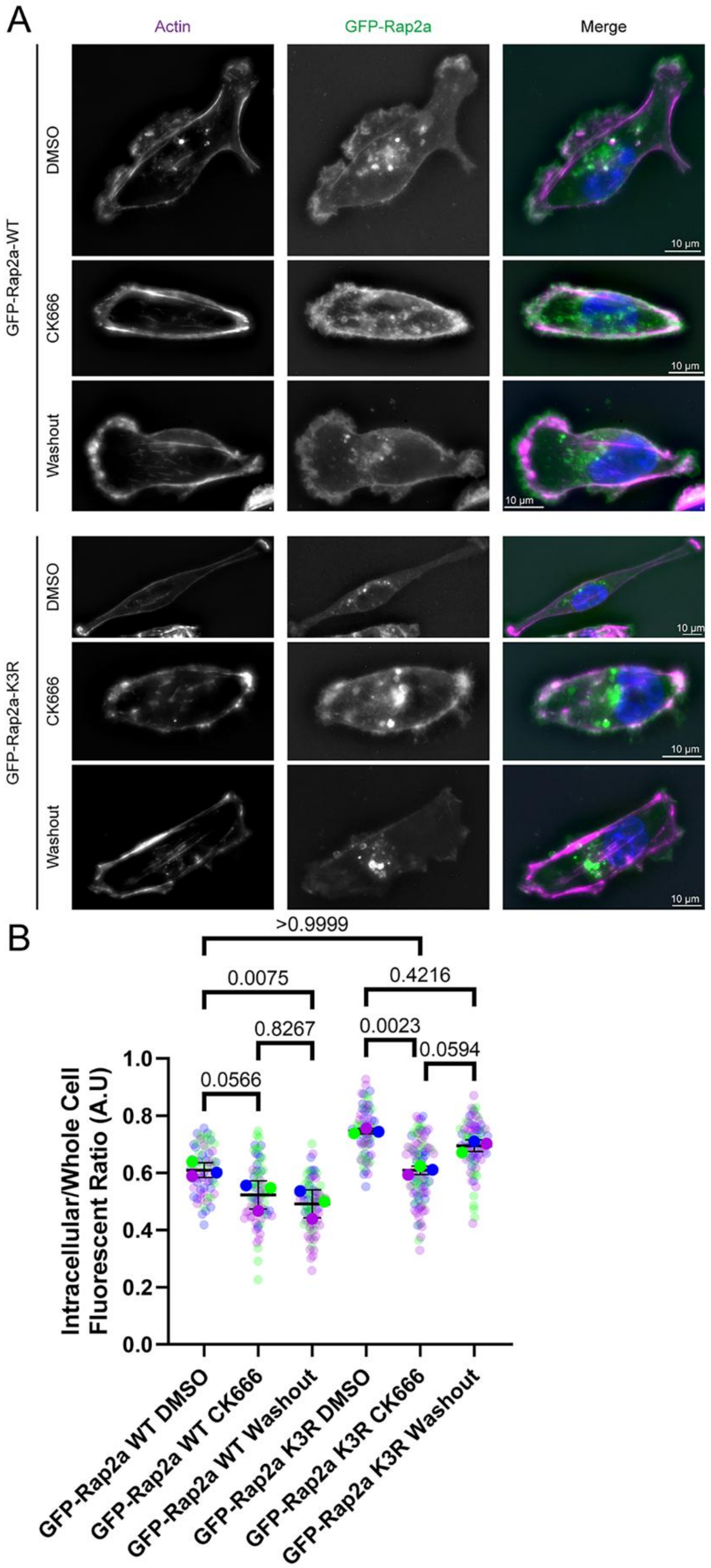


Journal of Cell Science • Supplementary information

**Fig. S4. Activity is necessary for Rap2 retention at lamellipodia membrane.** **(A-B)** MDA-MB-231 cells expressing either GFP-Rap2a-WT or GFP-Rap2a-K3R were treated with CK666 to inhibit macropinocytosis-dependent internalization of Rap2. CK666 was then washed out to resume the function of macropinocytic mechanisms. Quantification (n=3 biological replicates, GFP-Rap2a-WT DMSO= 66 total cells, GFP-Rap2a-WT CK666= 72 total cells, GFP-Rap2a-WT washout= 78 total cells, GFP-Rap2a-K3R DMSO=66 total cells, GFP-Rap2a-K3R CK666= 91 total cells, GFP-Rap2a-K3R washout=76 total cells. One-Way ANNOVA with multiple comparisons. Average is mean of biological replicates, errors bars represent biological replicate standard deviation. Significant P-values of vital comparisons shown) of Rap2 membrane localization is shown in **(B).**

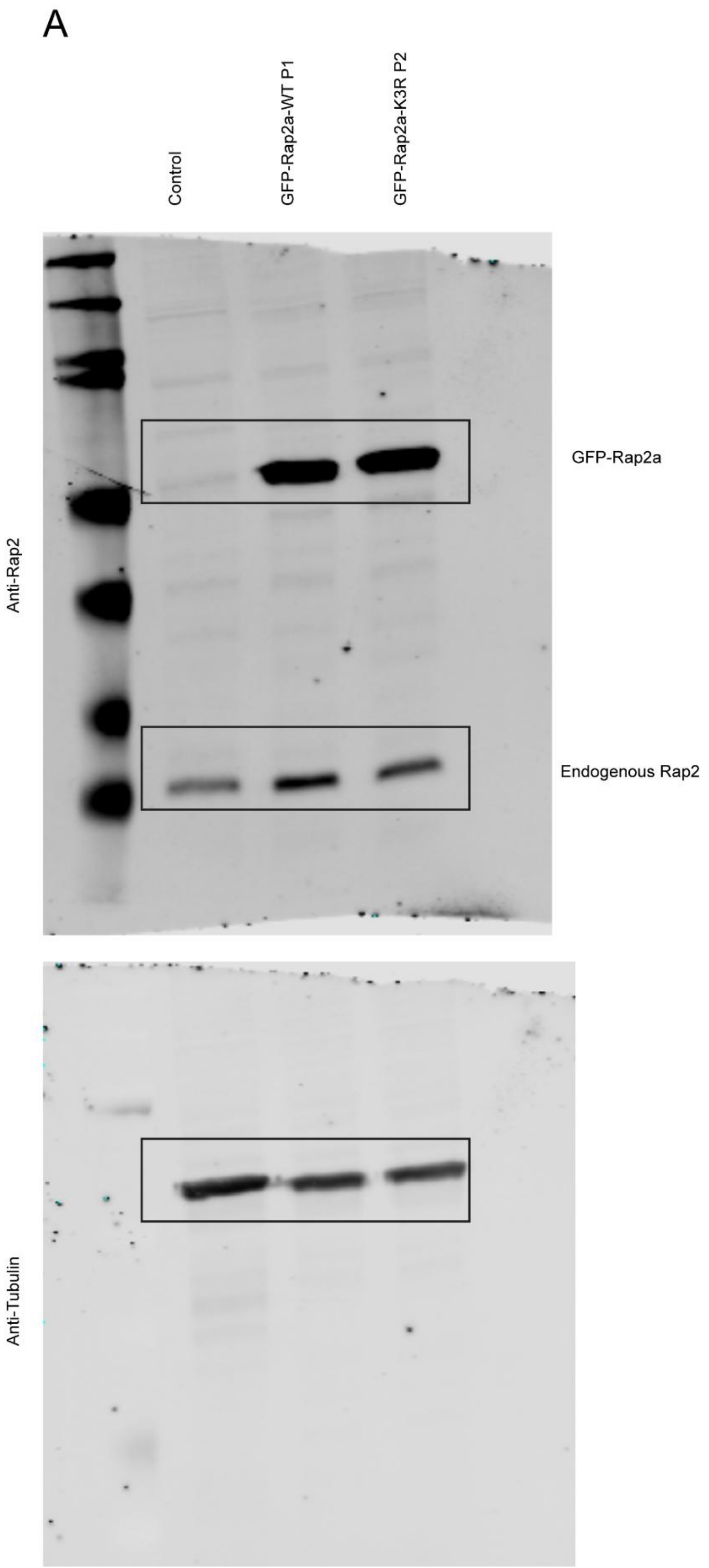


**Fig. S5. Western blots from figures 4 and 6. (A**) Full-length blot from Fig. S3E stained with Rap2 and tubulin.

Journal of Cell Science • Supplementary information

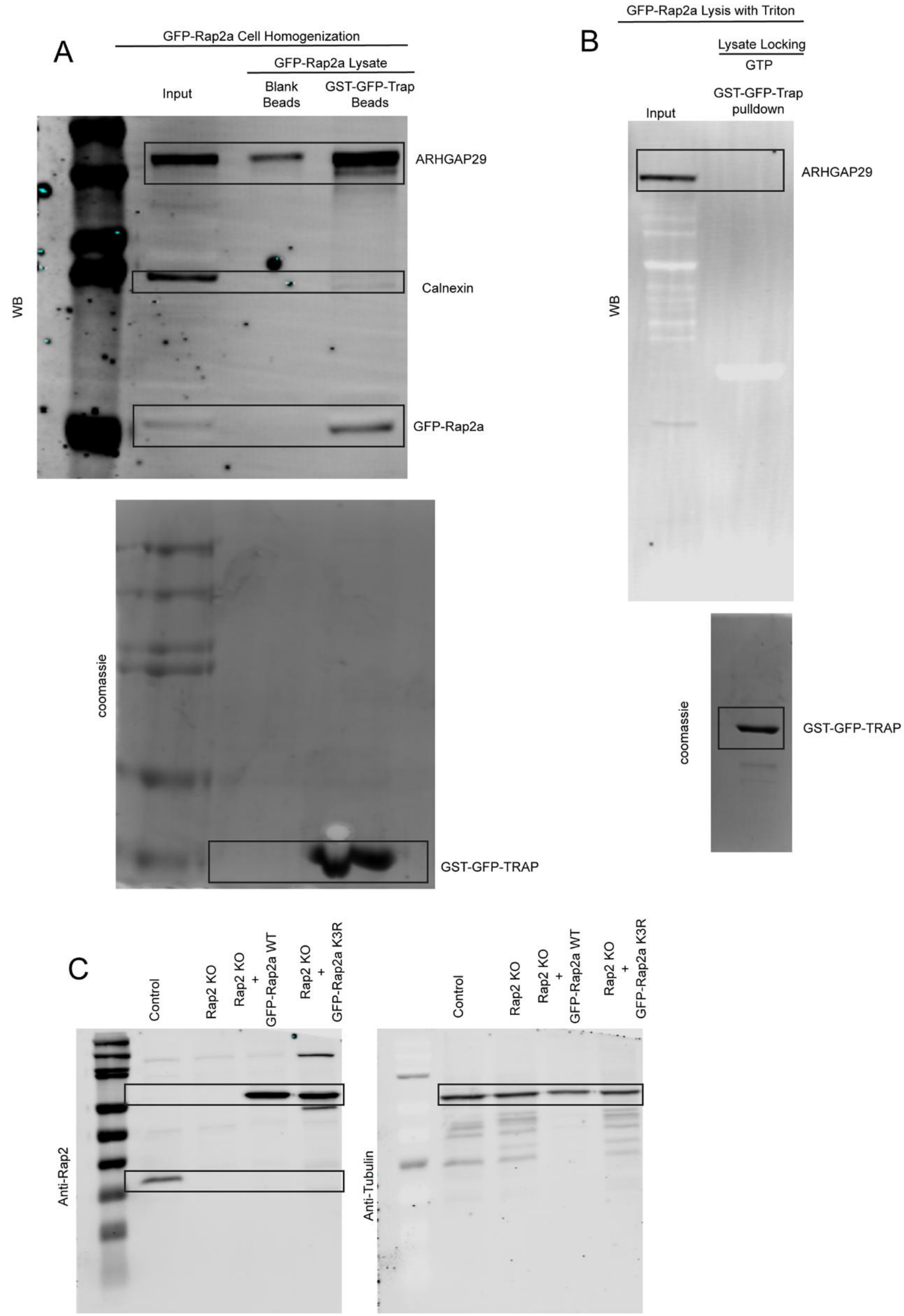


Journal of Cell Science • Supplementary information

**Fig. S6. Western blots from figures 4 and 6.**

**(A**) Full-length blot from Fig. 4G stained with ARHGAP29, Calnexin, and GFP and Coomassie for GST-GFP-TRAP.

**(B)** Full-length blot from Fig. 4H stained with ARHGAP29 and Coomassie for GST-GFP-TRAP.

**(C)** Full-length blot from Fig. 6I stained with Rap2 and tubulin.

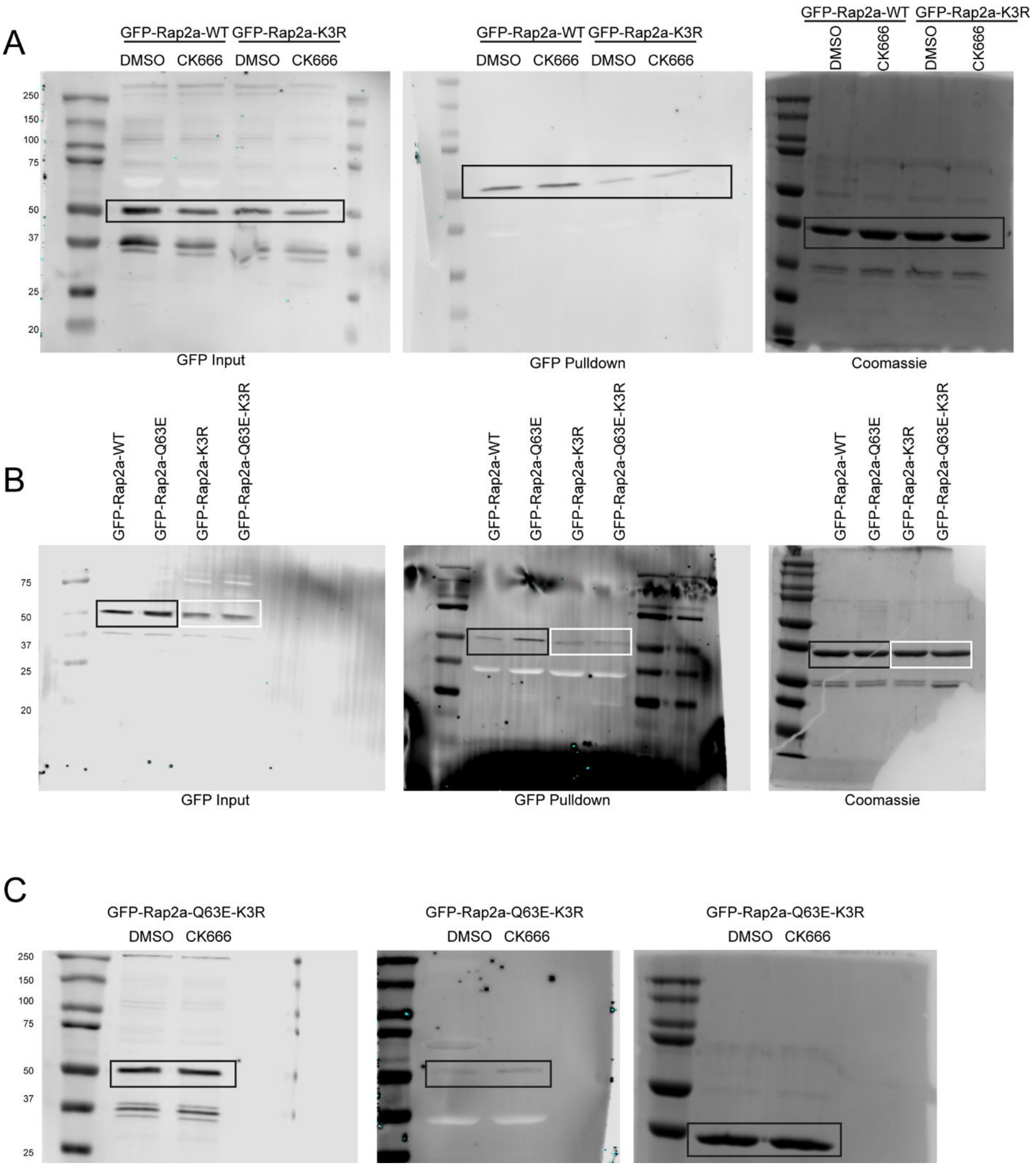


Journal of Cell Science • Supplementary information

**Fig. S7. Western blots from figures 8 and 9.**
**(A)** Full-length blot of Fig. 8G showing input and pulldown stained with GFP and Coomassie for GST-GFP-TRAP
**(B)** Full-length blot of 9C (black outline), and 9E (white outline) showing input and pulldown stained with GFP and Coomassie for GST-GFP-TRAP
**(C)** Full-length blot of Fig. 9G. input and pulldown stained with GFP and Coomassie for GST-GFP-TRAP

**Table S1. Antibodies and Reagents**

| Name | Company/Product Number | Dilution |
|---|---|---|
| RhoA | Sana Cruz, sc-26C4, mouse | 1:1000-1:500 |
| Rap2 | BD Biosciences 610215, mouse | 1:1000 |
| Tubulin | Li-Cor 926-42211, rabbit | 1:5000 |
| Cortactin | Cell Signaling, CST-3503, rabbit | 1:100 |
| ARHGAP29 | In house, rabbit | 1:100 |
| ARHGAP29 | Santa Cruz, sc-365554, mouse | 1:1000-1:500 |
| Calnexin | Transduction labs, C45520, mouse | 1:1000 |
| Phospho-Paxillin | Cell Signaling, CST-2541, rabbit | 1:50 |
| Myosin-IIB | Cell Signaling, CST-3404, rabbit | 1:100 |
| Ubiquitin P4D1 | Santa Cruz sc-8017, mouse | 1:100 |
| GFP | Invitrogen A-11122, rabbit | 1:100 |
| GFP | Proteintech, 1E10H7, mouse | 1:1000 |
| GDP | Abcam, ab146529 | N/A |
| Duolink in situ PLA probe | Sigma-Aldrich, DUO92002, rabbit (+) | 1:5 |
| Duolink in situ PLA probe | Sigma-Aldrich, DUO92004, mouse (-) | 1:5 |
| Duolink in situ detection reagents red | Sigma Aldrich, DUO92008, red | N/A |
| CK666 | Sigma-Aldrich, SML0006 | N/A |
| 8-bromo-cAMP | Tocris (biotechne), 1140 | N/A |
| X-tremeGENE 9 Transfection Reagent | Sigma-Aldrich, XTG9-RO | N/A |
| IRDye 680RD Anti-Mouse Secondary | Li-Cor 926-68072 | 1:5000 |
| IRDye 800CW Anti-Rabbit Secondary | Li-Cor 926-32213 | 1:5000 |
| Acti-stain 555 (phalloidin) | Cystoskeleton, PHDH1-A | 1:100 |
| Alexa 488 Anti-Rabbit secondary | Jackson ImmunoResearch 715-545-152 | 1:100 |
| Alexa 594 Anti-Mouse secondary | Jackson ImmunoResearch 715-585-150 | 1:100 |
| Alexa 594 Anti-Rabbit secondary | Jackson ImmunoResearch 711-585-152 | 1:100 |
| Hoechst 33342 | AnaSpec AS-83218 | 1:4000 |
| Bio-Rad Protein Assay Dye Reagent (Bradford method) | Bio-Rad 5000006 | 1:5 |
| Intercept TBS Blocking Buffer | Li-Cor 927-60001 | 1:3 |

Journal of Cell Science • Supplementary information

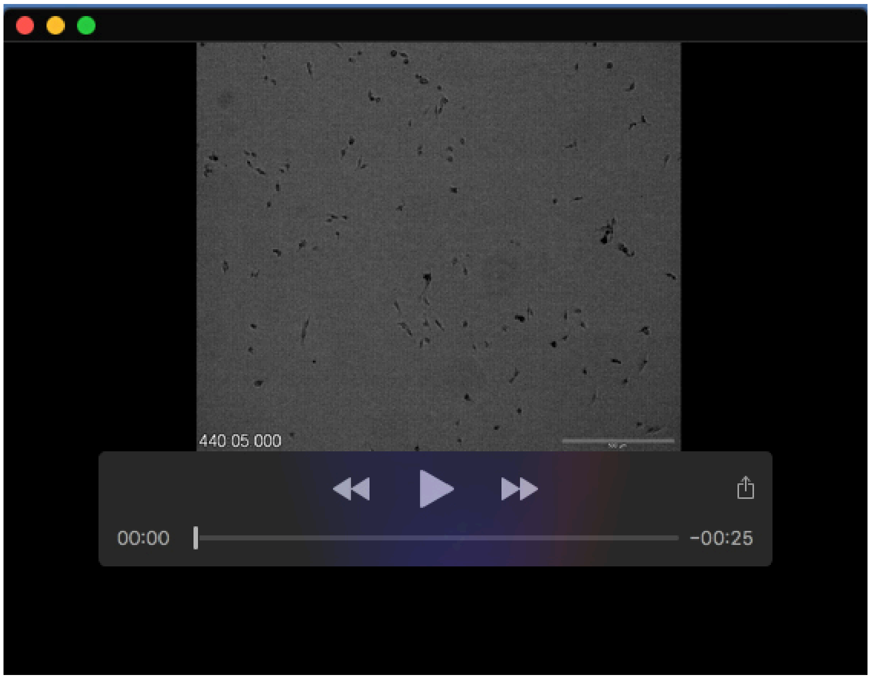


**Movie 1. Random migration assay of control cells.**
Control MDA-MB-231 cells were seeded on fibronectin coated plates imaged with the 4x objective of an OLYMPUS IX83 inverted confocal microscope. Imaging lasted 12 hours with images taken every 20 minutes.

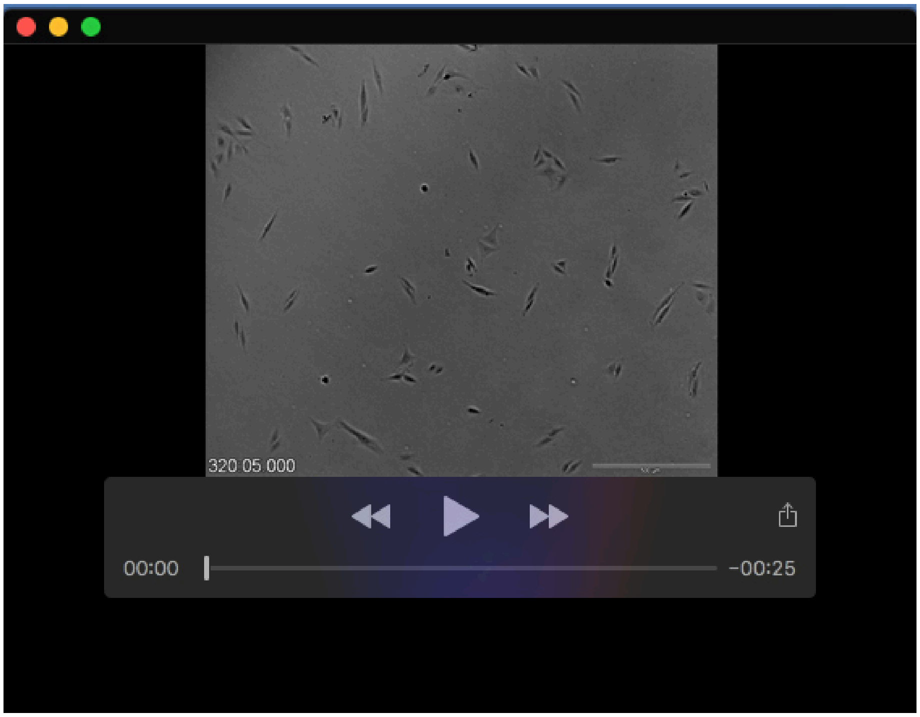


**Movie 2. Random migration assay of Rap2-KO cells.**
MDA-MB-231 cells were seeded on fibronectin coated plates imaged with the 4x objective of an OLYMPUS IX83 inverted confocal microscope. Imaging lasted 12 hours with images taken every 20 minutes.

Journal of Cell Science • Supplementary information

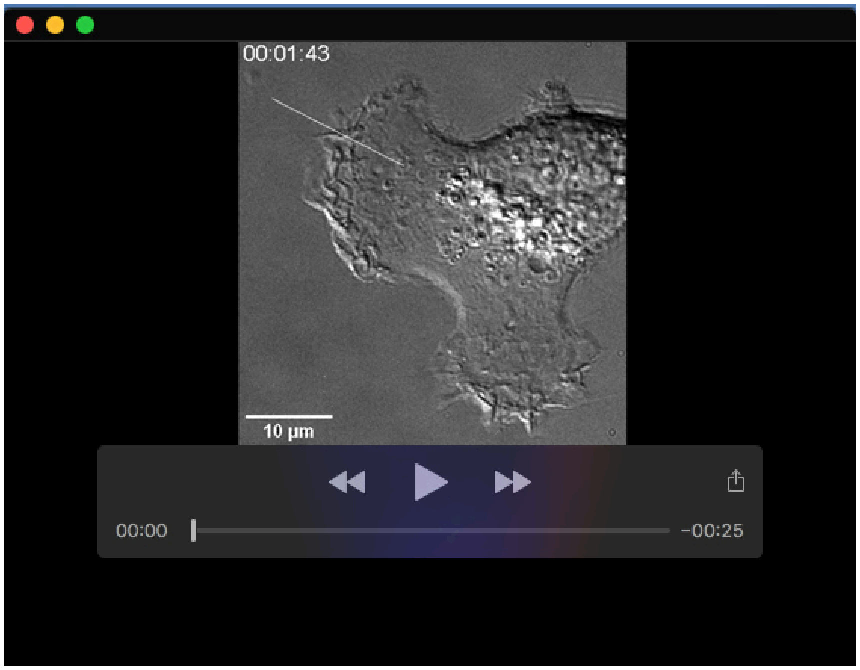


**Movie 3. Lamellipodium dynamics of control cells.**
Control MDA-MB-231 cells were seeded on collagen coated plates and imaged with the 63x objective using the DIC filter on a Zeiss 2 Axiovert 200M microscope. Images were taken every 1 second for 8 minutes. The white line represents where a kymograph was taken for SACED analysis.

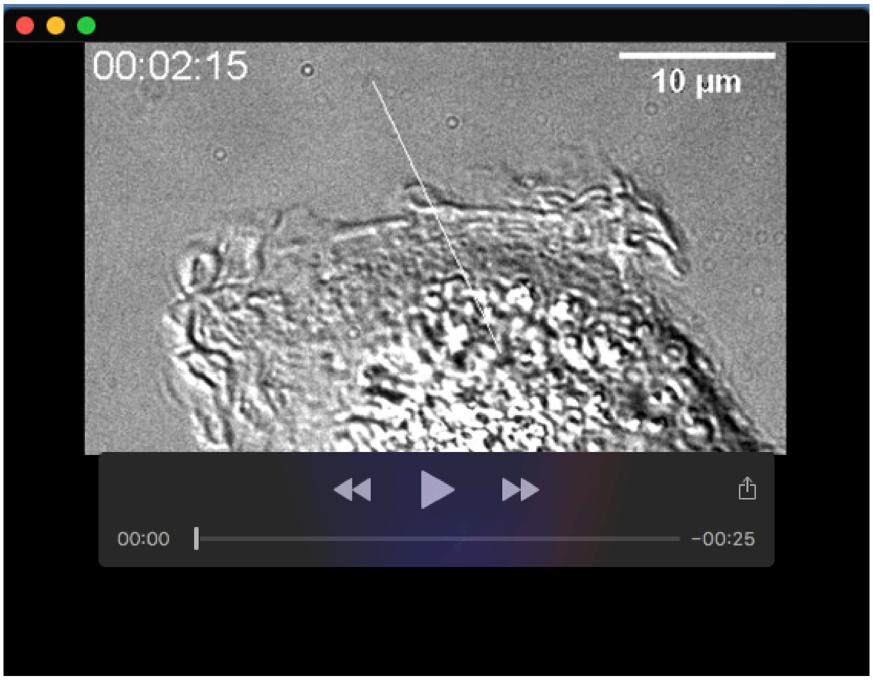


**Movie 4. Lamellipodium dynamics of Rap2-KO cells.**
Rap2KO MDA-MB-231 cells were seeded on collagen coated plates and imaged with the 63x objective using the DIC filter on a Zeiss 2 Axiovert 200M microscope. Images were taken every 1 second for 8 minutes. The white line represents where a kymograph was taken for SACED analysis.

Journal of Cell Science • Supplementary information

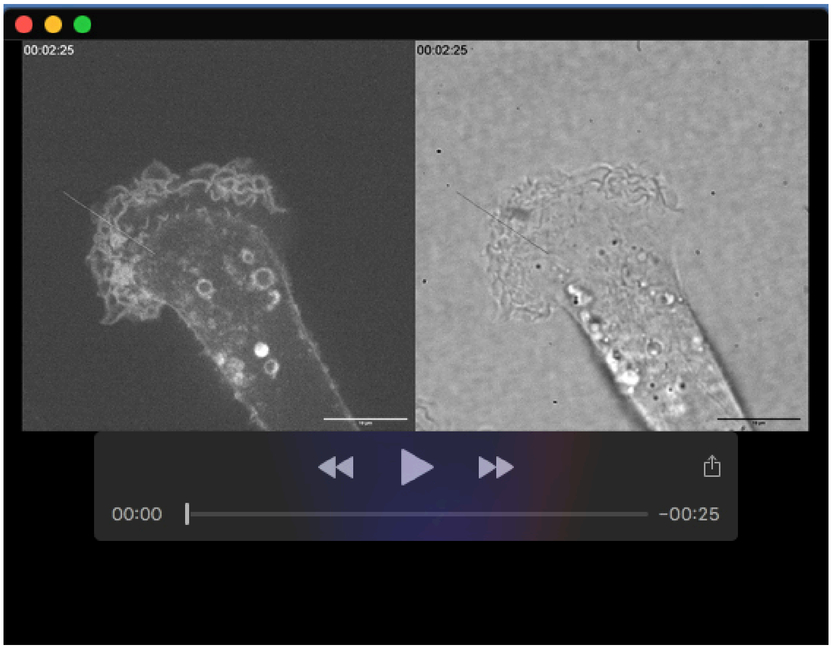


**Movie 5. GFP-Rap2a is enriched in retracting lamellipodia ruffles.**
MDA-MB-231 cells expressing GFP-Rap2a were seeded on collagen coated plates and imaged with the 100x objective and 2.8x CSU-W1 T1 Super-Resolution spinning disk confocal SoRa mag changer disk using the 488 (left panel) and transmitted light (right panel) channel on a Nikon Ti2-E inverted microscope. Images were taken every 5 seconds for 5 minutes.

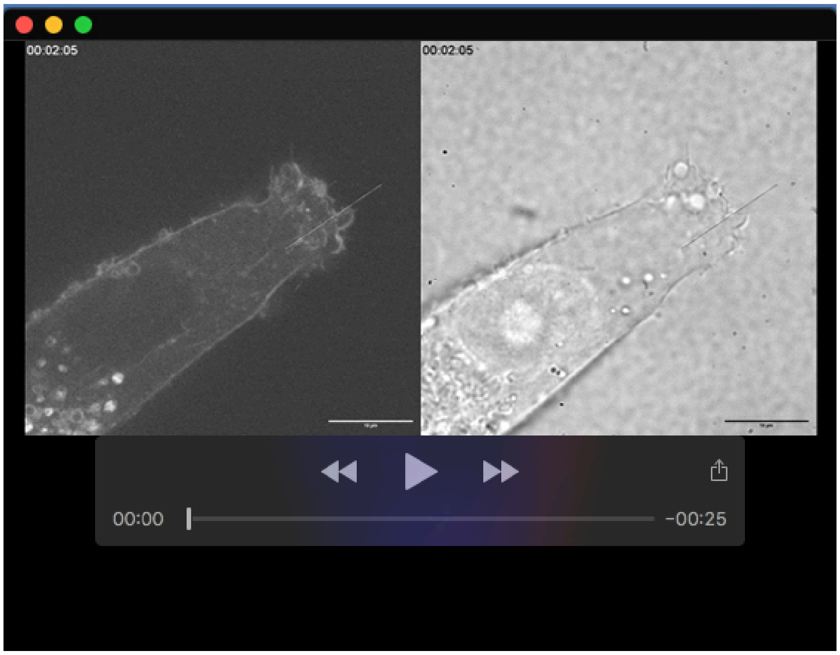


**Movie 6. GFP-Rap2a-Q63E overexpression alters lamellipodia dynamics.**
MDA-MB-231 cells expressing GFP-Rap2a-Q63E were seeded on collagen coated plates and imaged with the 100x objective and 2.8x CSU-W1 T1 Super-Resolution spinning disk confocal SoRa mag changer disk using the 488 (left panel) and transmitted light (right panel) channels on a Nikon Ti2-E inverted microscope. Images were taken every 5 seconds for 5 minutes.

Journal of Cell Science • Supplementary information

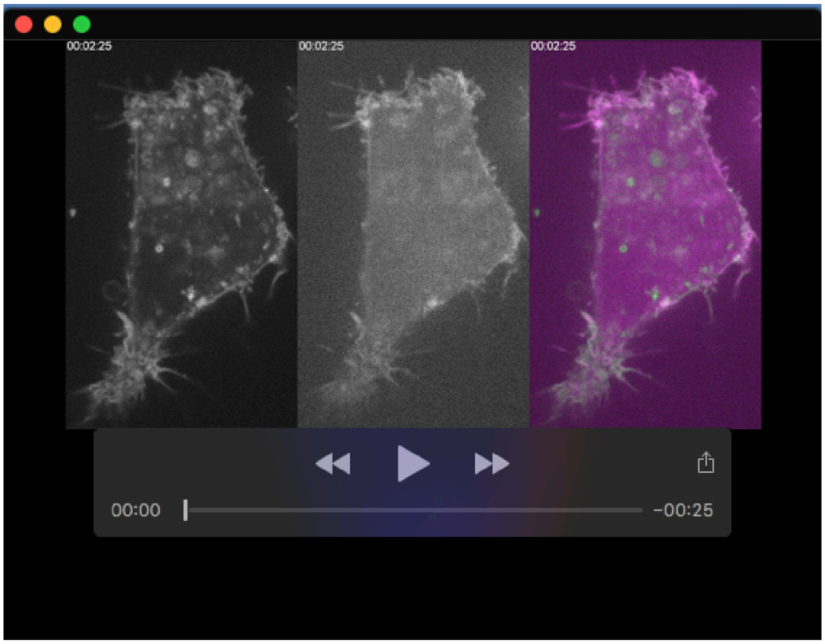


**Movie 7. GFP-Rap2a colocalizes with activated RhoA at the retracting lamellipodia ruffles.** MDA-MB-231 cells expressing GFP-Rap2a (green, left panel) were transfected with a dTom-2xrGBD RhoA biosensor (magenta, middle panel). Cells were seeded on collagen coated plates and imaged with the 100x objective and 2.8x CSU-W1 T1 Super-Resolution spinning disk confocal SoRa mag changer disk using the 488 and 596 channels on a Nikon Ti2-E inverted microscope. Images were taken every 5 seconds for 5 minutes.

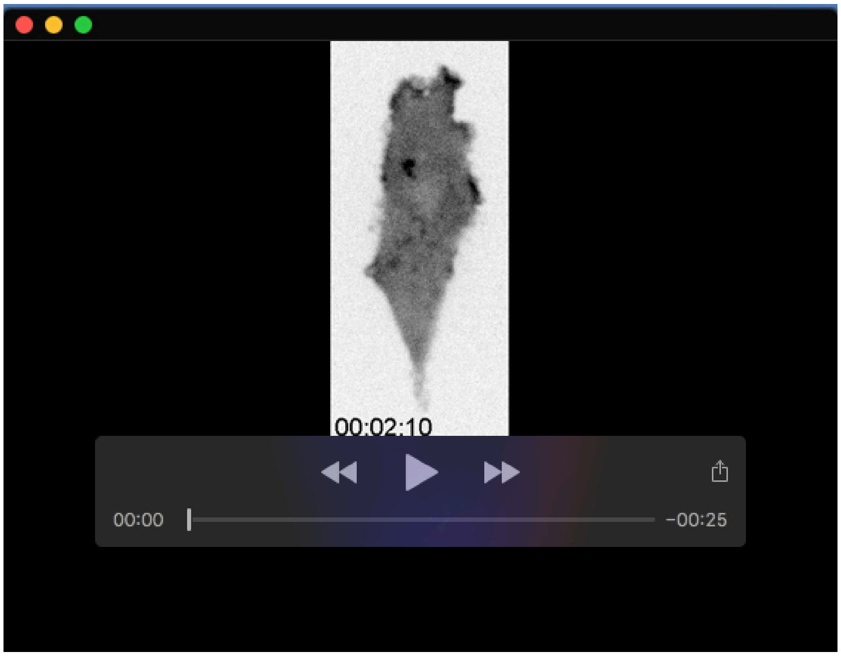


**Movie 8. RhoA biosensor dynamics in control cells.**
Control MDA-MB-231 cells were transfected with a dTom-2xrGBD RhoA biosensor. Cells were seeded on collagen coated plates and imaged with the 100x objective and 1.0x CSU-W1 T1 Super-Resolution spinning disk confocal SoRa mag changer disk using the 596 channels on a Nikon Ti2-E inverted microscope. Images were taken every 5 seconds for 5 minutes.

Journal of Cell Science • Supplementary information

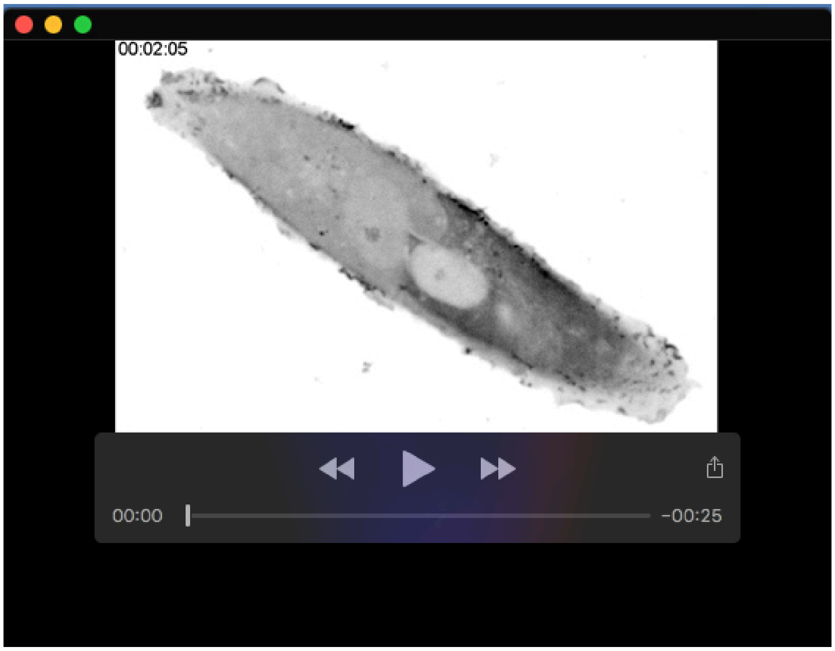


**Movie 9. RhoA biosensor dynamics in Rap2-KO cells**

Rap2-KO MDA-MB-231 cells were transfected with a dTom-2xrGBD RhoA biosensor. Cells were seeded on collagen coated plates and imaged with the 100x objective and 1.0x CSU-W1 T1 Super-Resolution spinning disk confocal SoRa mag changer disk using the 596 channels on a Nikon Ti2-E inverted microscope. Images were taken every 5 seconds for 5 minutes.

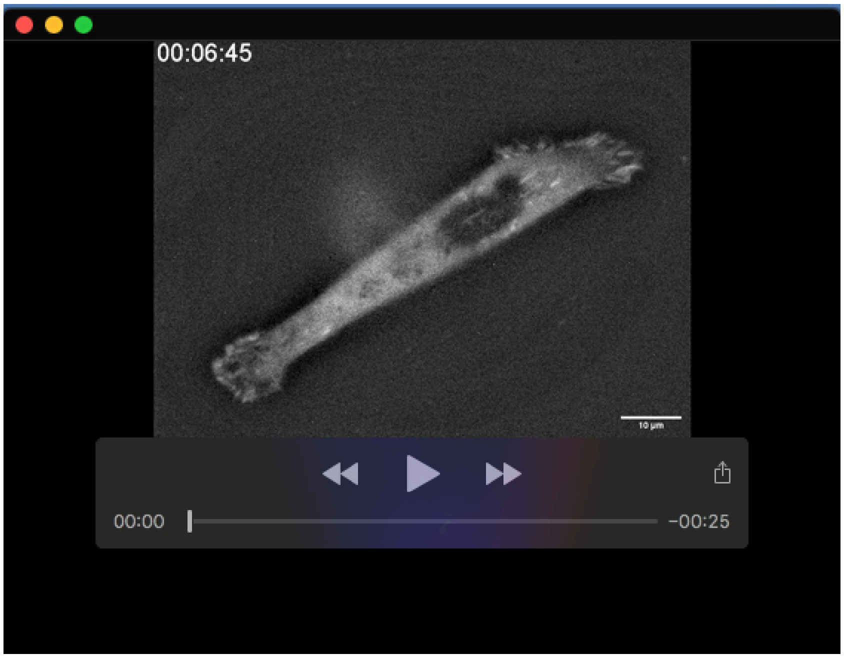


**Movie 10. Focal adhesion dynamics in control cells.**

Control MDA-MB-231 cells were transfected with a GFP-Paxillin constructs and seeded on collagen coated plates. Cells were imaged with the 63x objective using the 488 filter on a Zeiss 2 Axiovert 200M microscope. Images were taken every 15 seconds for 30 minutes. No neighbors deconvolution was performed.

Journal of Cell Science • Supplementary information

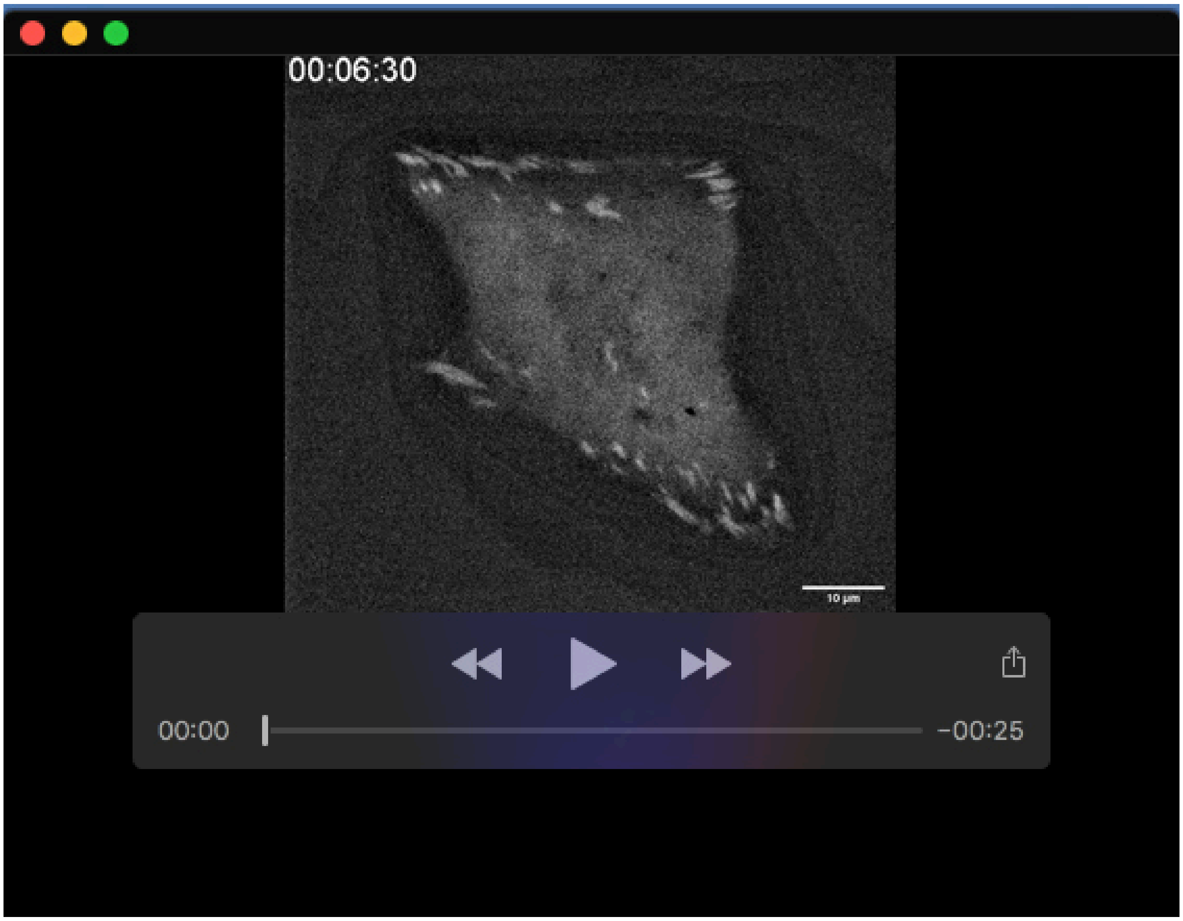


**Movie 11. Focal adhesion dynamics in Rap2-KO cells.**

Rap2KO MDA-MB-231 cells were transfected with a GFP-Paxillin constructs and seeded on collagen coated plates. Cells were imaged with the 63x objective using the 488 filter on a Zeiss 2 Axiovert 200M microscope. Images were taken every 15 seconds for 30 minutes. No neighbors deconvolution was performed.
